# Analyze and Identify Peiminine Target EGFR Improve Lung Function and Alleviate Pulmonary Fibrosis to Prevent Exacerbation of Chronic Obstructive Pulmonary Disease by Phosphoproteomics Analysis

**DOI:** 10.3389/fphar.2019.00737

**Published:** 2019-07-03

**Authors:** Xiaoyao Ma, Aina Liu, Wenjuan Liu, Zhihua Wang, Nianwei Chang, Suyun Li, Jiansheng Li, Yuanyuan Hou, Gang Bai

**Affiliations:** ^1^State Key Laboratory of Medicinal Chemical Biology, College of Pharmacy and Tianjin Key Laboratory of Molecular Drug Research, Nankai University, Tianjin, China; ^2^Key Laboratory of Hormones and Development (Ministry of Health), Tianjin Key Laboratory of Metabolic Diseases, Metabolic Diseases Hospital and Tianjin Institute of Endocrinology, Tianjin Medical University, Tianjin, China; ^3^Tianjin University of Traditional Chinese Medicine, Tianjin, China; ^4^First Affiliated Hospital of Henan University of Traditional Chinese Medicine, Zhengzhou, China

**Keywords:** phosphoproteomics, peiminine, EGFR, chronic obstructive pulmonary disease, lung function

## Abstract

Chronic obstructive pulmonary disease (COPD) has been a major public health problem and is still a formidable challenge for clinicians. It is urgent to find new compounds for minimizing the risk of disease progression and exacerbation especially in the early phase of COPD. A traditional Chinese medicine (TCM) formula, Chuan Bei Pi Pa dropping pills (CBPP), was tested in this study to investigate its potential mechanisms in preventing the exacerbation of COPD. Phosphoproteomics analysis for a smog stimulated early stage COPD mice model was employed to detect the underlying molecular mechanisms of CBPP. In addition, protein–protein interaction (PPI) and bioinformatics analyses were included to analyze the key proteins and predict the key bioactive compounds. The results indicated that peiminine (PEI) target epidermal growth factor receptor (EGFR) prevented the exacerbation of COPD by inhibiting the EGFR signaling pathway, and ursolic acid (UA) can alleviate inflammation disorders *via* inhibition of CASP3 on mitogen-activated protein kinase (MAPK) signaling pathway. After *in vivo* and *in vitro* evaluations, we revealed that PEI from CBPP, as a lead compound, can improve lung function and alleviate pulmonary fibrosis by acting on the EGFR and MLC2 signaling pathways. Furthermore, the approach described here is an effective way to analyze and identify the bioactive ingredients from a mixture by functional proteomics analysis.

## Introduction

Air pollution has become a major global problem in developing countries, particularly those with rapid industrialization (Beran et al., 2015). Only 12% of urban population meet the World Health Organization air quality standards (Norebert, 2016). Air pollution has been affirmed to be related to the mechanisms of compromised pulmonary immune defense in animals and humans. Both acute and chronic living in air pollution contribute to health risks, which can cause from slight stimulation of the upper respiratory system to chronic respiratory diseases [chronic obstructive pulmonary disease (COPD)], e.g., asthma and lung cancer (Hoffmann et al., 2012; Ko and Hui, 2012; Wellenius et al., 2012).

COPD has been a main public health problem and remains a formidable challenge for clinic treatment. Because of the high incidence rate and fatality rate of COPD, it has made challenges for human health (López-Campos et al., 2016). The most common drugs for the treatment of COPD are muscarinic antagonists, beta-agonists, and inhaled corticosteroids (Tashkin and Fabbri, 2010). The severity of COPD is divided into four stages: mild, moderate, severe, and very severe. Maintenance therapy with drugs is used in patients with stage II to IV COPD. However, current drug treatments for COPD only have the effect of inhibiting chronic inflammation, and it can’t reverse the course of disease or prevent exacerbation of COPD (Mohamed Ismail et al., 2018). To minimize the risk in the course of disease, exacerbation is the key in stage I of COPD, but there are no effective means to achieve this goal (Singh et al., 2016). Hence, it is necessary to develop a novel composite drug for treatment in stage I COPD to improve bronchodilation and lung function and alleviate inflammation, thereby preventing the exacerbation of COPD.

Traditional Chinese medicine (TCM) plays a significant role in the therapeutic process of chronic diseases (Changxiao, 2018). In the clinic, Yupingfeng granules alleviated the exacerbations of stage II to III and increased the condition in stage II to IV of COPD (Ma et al., 2018). Hence, it is possible to find promising compounds from TCM formulas. Chuan Bei Pi Pa dropping pills (CBPPs) are a traditional format and have been used for eliminating inflammation of the lung and improving lung function (Jin Tao et al., 2016). Therefore, CBPP has the potential to prevent COPD by alleviating the stimulation of air pollution in the early phase. CBPP is composed of five herbal medicines, which are loquat leaf (*Eriobotrya japonica Thunb*), fritillary (*Fritillaria*), Pinellia ternata (*Pinellia ternata*), balloon flower (*Platycodon grandiflorus*), and oil extracts from mint (*Mentha haplocalyx Briq*). The chemical studies carried out by UPLC/Q-TOF MS revealed that the primary components are pentacyclic triterpenoids and alkaloid (Jin Tao et al., 2016). Although the prediction of the mechanisms of action of these compounds had been accomplished by network pharmacology, it has not been verified.

In nature, the phosphorylation of protein is one of the main regulatory mechanisms. Phosphorylation is a key regulator in biological processes of cells and is involved in the regulation of diverse processes. Rapid advances in phosphoproteomics analysis have opened new avenues for analyzing and identifying the bioactive nutrients *in vivo* (Girolamo and Petsalaki, 2017). These analyses allow us to detect the alteration of protein phosphorylation levels and analyze the potential mechanisms by which compound mixtures act by phosphoproteomics analysis. This analysis provides the possibility to continue to find promising compounds. The worth of phosphoproteomics analysis has been demonstrated to provide the information of mechanisms in diseases and elucidating the mechanisms of action of bioactive compounds in many studies (Locard-Paulet et al., 2016; Wei et al., 2016). Phosphoproteomics analysis has been used to find the mechanism of resistance of melanoma cells to serine/threonine-protein kinase B-raf (BRAF) inhibitors, which increased MAPK10 phosphorylation and regulated the key substrates in the Rho/ROCK signaling axis, providing basis for the novel combination therapy with an mTOR inhibitor for the latter (Parker et al., 2015). In addition, phosphoproteomics data suggest that resveratrol inhibits autophagy in serum-deprived cells by decreasing the phosphorylation of PRAS40^T246^ and PRAS40^S183^ and increasing the binding of PRAS40 to RAPTOR/TORC1 in the mTORC1 signaling pathway (Alayev et al., 2014).

In this study, we evaluated the effects of CBPP treatment in mice exposed to smog stimulus as a model of early stage of COPD. We employed the phosphoproteomics analysis method to detect the alterations in protein phosphorylation levels in lung tissues, to identify the potential targets, and to provide the means to prevent the exacerbation of COPD. Then, we analyzed the phosphorylated proteins by bioinformatics methods and detected the effects of the main compounds on inflammation, cell contraction, and fibrosis-related signaling pathways by molecular biological methods. The results demonstrated the potential of the bioactive compounds from CBPP to act as therapeutics for the prevention of COPD by alleviating inflammation and improving lung function in exacerbation of COPD.

## Materials and Methods

### Reagents and Materials

CBPP (lot no. 635031) was donated by no. 6 TCM Factory of Zhongxin Pharmaceuticals (Tianjin, China). The quality of each herb was verified by marker compounds. Peiminine [PEI; purity > 98%, determined by high-performance liquid chromatography (HPLC)] was purchased from Aladdin (Shanghai, China). Chemiluminescent HRP substrates were purchased from Millipore Corporation (MA, USA). Primary antibodies anti-MLC2 (#3627), anti-phospho-MLCS19 (#3671), anti-ERK1/2 (#9102), anti-NF-kB (#8242), anti-phospho-ERK1/2 (#4370), anti-phospho-NF-kB (#3033), anti-β-Actin (#4970), anti-GADPH (#2118), and a goat anti-rabbit IgG secondary antibody (#7074) were purchased from Cell Signaling Technology (Beverly, MA, USA). Anti-ROCK1 (ab45171), anti-phospho-ROCK1T455+S456 (ab203273), anti-AKT (ab39364), and anti-phospho-AKTS473 (ab81283) were purchased from Abcam (Cambridge, UK). All the reagents used in cell culture were purchased from Gibco BRL Life Technologies (Grand Island, NY, USA).

### Animal Experiments and Sample Preparation

Male Kunming mice (18–22 g) were purchased from the Experimental Animal Center of the National Institute for the Control of Pharmaceutical and Biological Products (Beijing, China, lot no. 0006407). The animals were housed in a suitable environment and were free to get food and water. The mice adjusted to the environment in 3 days. These mice were divided into six groups (n = 12) randomly: the control group (Con), the model group (Mod), the positive dexamethasone group (Dex, 0.2 mg/kg daily), and different CBPP dose treatment groups (200 mg/kg CBPP-H, 100 mg/kg CBPP-M, and 50 mg/kg CBPP-L). Then, the mice were exposed to a mixture of SO_2_ (60–120 PPM) and heavy smog from the cigarette (approximately 2000 PM2.5) twice a day for half an hour at a time. The entire process was continued for 45 days. On the 10th day of exposure, the drugs were given intragastrically to each group for the following 5 weeks. Con and Mod received the same volume of saline.

After 45 days, retro-orbital blood samples were taken from the mice. The blood was centrifuged at 4,000 r/min for 5 min, and serum supernatant was used in the testing. Then, the animals were euthanized, and the right lungs were ligated. To prepare the bronchoalveolar lavage fluid (BALF), the left lung was washed with 0.9 ml of phosphate buffer saline (PBS) three times. The BALF was centrifuged and the supernatant was collected for cytokine analysis. The cell precipitate was resuspended in PBS (100 ml) for leukocyte counts. The inflammatory cytokines, such as TNF-α and IL-8, in the serum were measured by ELISA kits. Then, the right lung was fixed in 4% paraformaldehyde 24 h and cut into slices for hematoxylin and eosin (H&E) staining and Masson staining. The left lung was rapidly stored at −80°C for isotope techniques of relative labeling and absolute quantification (iTRAQ) quantitation phosphoproteomics analysis.

### Protein Extraction, Digestion, and iTRAQ Quantitation Phosphoproteomics Analysis

Six lung tissue samples, including samples from the Con, Mod, and CBPP-H groups, were chosen for iTRAQ-based quantitation phosphoproteomics analysis. Each group had two biological repetitions, marked 1 and 2. First, protein extracts and quality control using Bradford and SDS-PAGE were performed. Second, the protein was digested to peptides with trypsin. Then, the peptides were labeled with different iTRAQ-8 plexes. Con1 and Con2 were labeled with iTRAQ 114 and 115, Mod1 and Mod2 with iTRAQ 116 and 113, and CBPP1 and CBPP2 with 119 and 121. The peptides were purified and enriched using TiO_2_ chromatography. Then, a set of tandem HPLC and Q-Exactive mass spectrometry (MS) was established for the qualitative assay. Proteome Discoverer 1.4 (Thermo Fisher) in combination with Mascot 2.3 analysis software was used to analyze MS data and to identify phosphorylation sites and peptides. The screened peptides were compared to each group, such as Con vs. Mod, CBPP vs. Mod, and CBPP vs. Con. The division value of the differential expression quantity of two groups was shown as the Log2 ratio. Cluster analysis was implemented with MATLAB 2011a (MathWorks, USA).

### Bioinformatics Analysis

To identify the pathways, we used the UniProt-GOA database, Gene Ontology (GO) annotation data, and kyoto encyclopedia of genes and genomes (KEGG) database. To analyze the significant difference pathways, the data were corrected as p-value < 0.05. Then, the pathways were classified according to the KEGG. The protein–protein interaction (PPI) and function protein analysis were used to identify the protein function and interaction. The details of this experiment are reported in our previous work (Wang et al., 2019). In molecular docking, we used Protein Data Bank (PDB), SYBYL 2.0 software, AutoDock 4.2, and Pymol in the analysis. The details of this experiment are reported in our previous work (Ma et al., 2017).

### Cell Culture and Western Blot

The BEAS-2B cell line was cultured in 1640 supplemented with 1% penicillin-streptomycin and 10% fetal bovine serum. Human bronchial smooth muscle cells (HBSMCs) were cultured with complete F12-DMEM with 1% penicillin-streptomycin and 10% fetal bovine serum. All cells were incubated in a humidified incubator at 37°C in a 5% CO_2_ atmosphere. The Western blot analysis protocol was as reported in our previous study (Ma et al., 2017).

### Statistical Analysis

The results were shown as the mean ± standard deviation (SD). For single comparisons, significant differences between the means were determined using Student’s *t*-test. A p-value less than 0.05 was considered to indicate significant differences. All data were processed using GraphPad Prism 5.01 software.

## Results

### Smog Stimulus-Induced Inflammation Alleviated by CBPP in Mice

**Figure 1:**
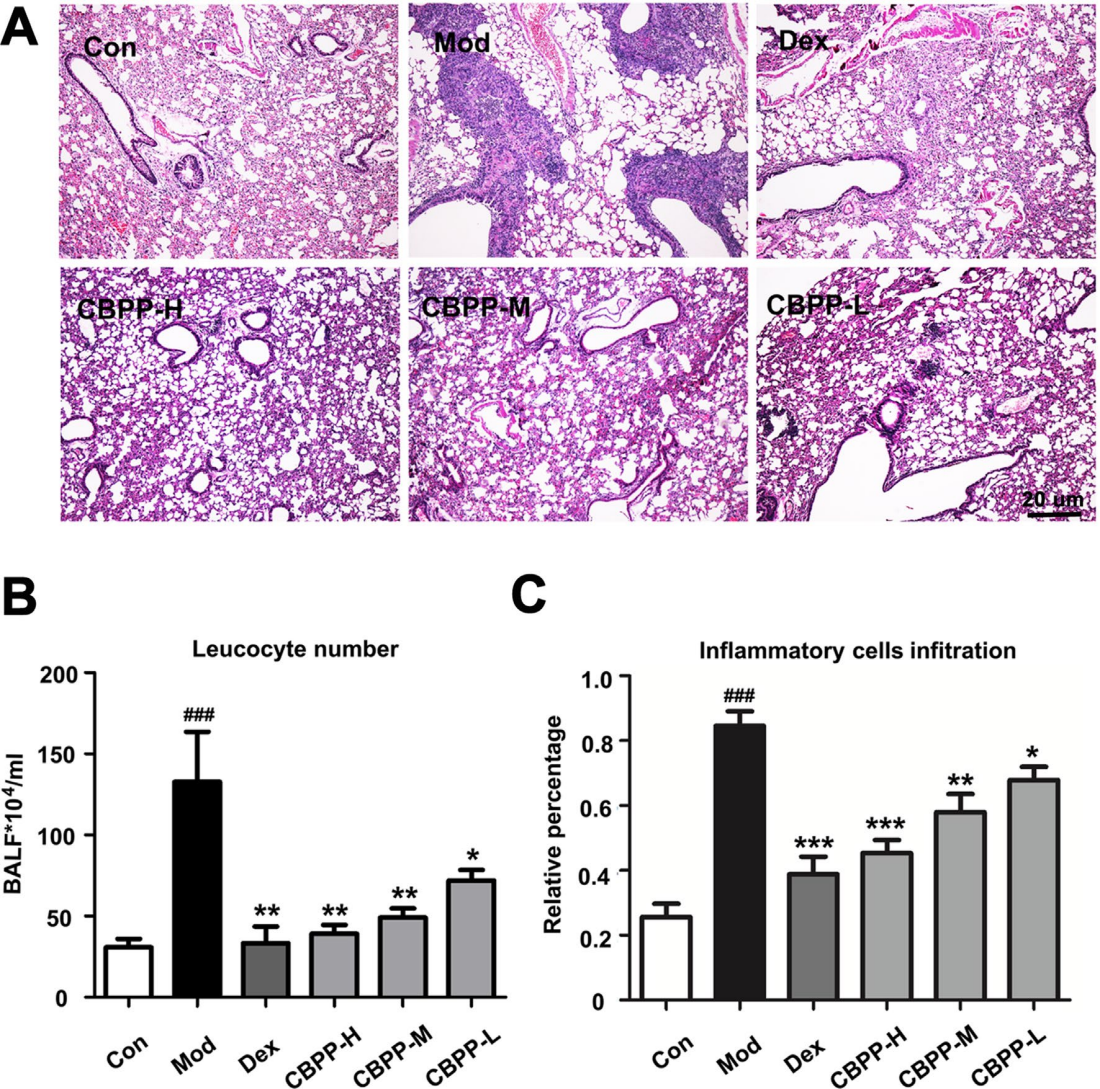
CBPP alleviated smog stimulus-induced inflammation in mice. **(A)** HE staining images (40-fold) of mice lungs. **(B)** Leukocyte numbers in BALF. **(C)** Inflammatory cell infiltration in mice lungs. *p < 0.05 and **p < 0.01 and ***p < 0.001 compared to the Mod. ^###^ *p* < 0.001 compared to the Con (n = 6).

To evaluate the therapy effects of CBPP on smog stimulus-induced COPD in mice, the changes of lung histology, leukocyte number, and inflammatory cells infiltration were tested. As shown in **Figure 1A**, smog stimulation caused capillary congestion, as well as the obstruction of the small airways by a large number of acute or chronic inflammatory cell infiltrates, which were compared to the Con group. Meanwhile, the treatment with CBPP alleviated the lung injury and decreased the obstruction of the recruitment of inflammatory infiltrate and the small airways significantly (**Figure 1B**). As shown in **Figure 1C**, the number of leukocytes was significantly increased in the Mod group, which was compared with the Con group. However, the leukocytes were decreased after the treatment of CBPP in a dose-dependent manner. The results suggested that CBPP alleviated the injury and inflammation in the lung tissues.

### ITRAQ-Based Quantitation Phosphoproteomics Analysis of Lung Tissues

**Figure 2:**
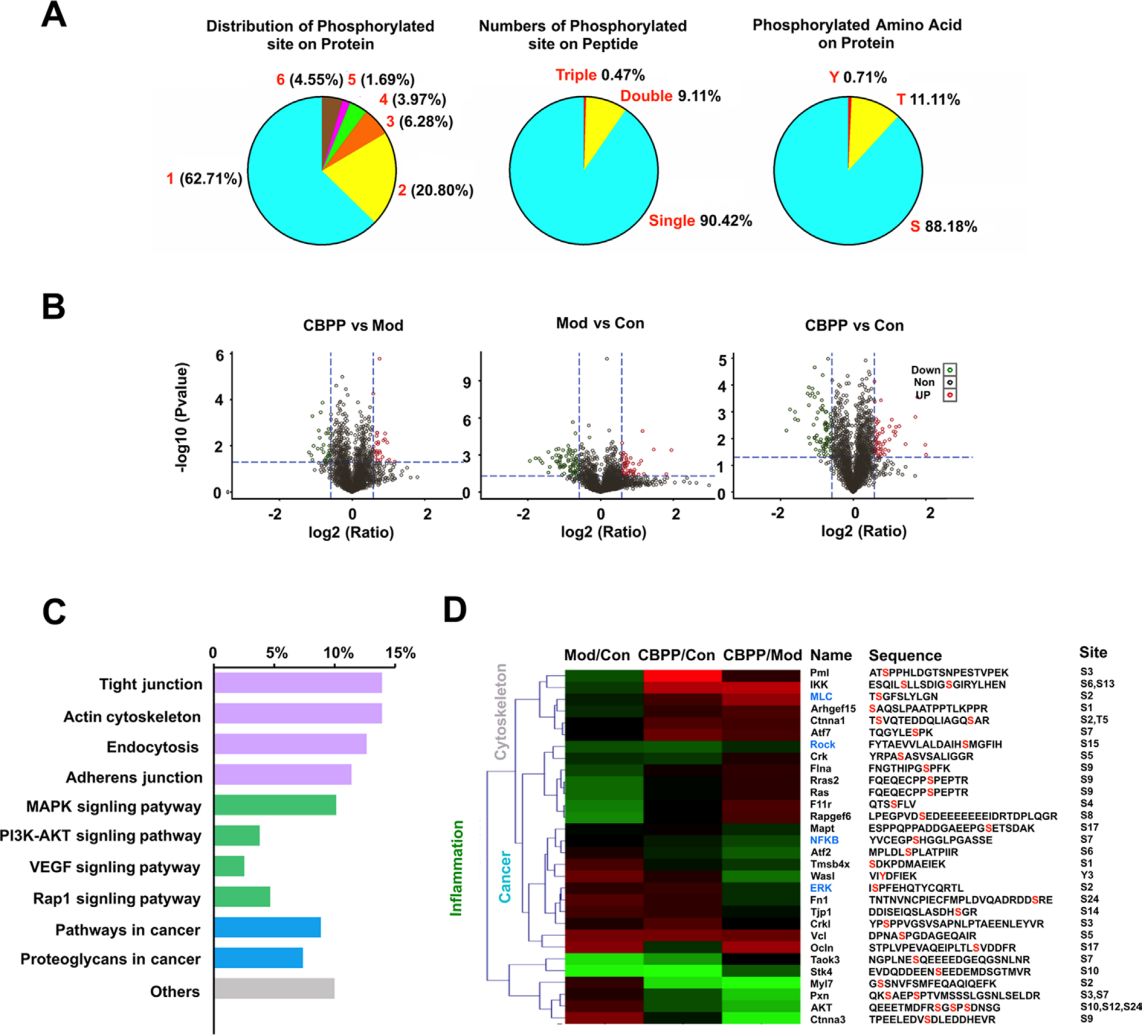
ITRAQ-based quantitation phosphoproteomics analysis of lung tissue. **(A)** Identification of phosphoproteome and displayed the information of phosphorylation of the protein, peptide, and amino acid. **(B)** Quantitation of phosphoproteome in volcano plots, satisfy multiple differences log2 ratio ≤ 1.3 or ≥0.667 and P-value ≤0.05; red dots: upregulated; green dots: downregulated. **(C)** The analysis of signaling pathways and function of significant phosphorylated peptides. **(D)** The heat map analysis of proteins related with inflammation, cancer, and the cytoskeleton, as well as information of identified phosphorylated peptide.

For exploring the underlying molecular mechanisms, an iTRAQ-based quantitation phosphoproteomics strategy was used. The Q-Exactive mass spectrometer (Thermo Fisher) was used for protein detection; the Proteome Discoverer analysis software (Thermo Fisher) was applied for further data analysis, and a musculus corresponding protein sequence database (76,576 protein sequences) was used as the searching database; 74,874 spectra maps were produced. With the condition of a false discovery rate (FDR) ≤ 0.01, 11,392 spectra maps were identified. Overall, 2,546 phosphorylated peptides and 2,116 phosphorylation sites (phosphors probability ≥ 0.75) were distributed on 1,120 phosphorylated proteins. On the protein level, there were commonly one to three phosphosites on each protein; on the peptide level, most had only single (90.42%) or double (9.11%) phosphosites. Additionally, protein phosphorylation usually occurred in serine (S) or threonine (T) or tyrosine (Y), which are involved in different biological processes. Most of the phosphosites were distributed on S (88.18%) and T (11.11%), with only 0.71% on Y (**Figure 2A**). Differential levels of phosphorylated proteins were screened under the standards of Control vs. Model (632 phosphorylated peptides), CBPP vs. Model (520 phosphorylated peptides), and CBPP vs. Control (554 phosphorylated peptides) according to log2 ratio ≤ 1.3 and log2 ratio ≥ 0.667. The up- or downregulated phosphorylated peptides are shown in red and green, respectively, in the volcano plots (**Figure 2B**).

### The GO and KEGG Pathway Annotation of Phosphorylated Proteins

GO is an international standardization of the gene function classification system, which has three ontologies that describe the molecular function and cellular component and biological process. As a result, a total of 42 significant phosphorylated peptides were screened and then clustered according to log2 ratio. Hence, the corresponding proteins of the 42 significantly different phosphorylated peptides were analyzed in the GO functional annotation.

These phosphorylated proteins were analyzed by the KEGG database. A total of 10 KEGG pathways were annotated, which can be divided into four categories. As shown in **Figure 2C**, the functions mainly have inflammation (green), cancer (blue), cytoskeleton (gray), and others. Meanwhile, four pathways were related to the actin cytoskeleton, four pathways were related to inflammation, and four pathways were related to cancer. Of the 42 phosphorylated proteins, 30 proteins were associated with the actin cytoskeleton, inflammation, or cancer. We focused on the pathways of the cytoskeleton and inflammation, which are the main biological processes of COPD. MLC2 and ROCK were associated with the actin cytoskeleton; ERK and NF-κB were associated with inflammation that was found in the phosphorylated proteins (**Figure 2D**, blue). The results suggest that CBPP can improve the state of lung cells by regulating the cytoskeleton and inflammation.

### PPI Analysis and Molecular Docking

**Figure 3:**
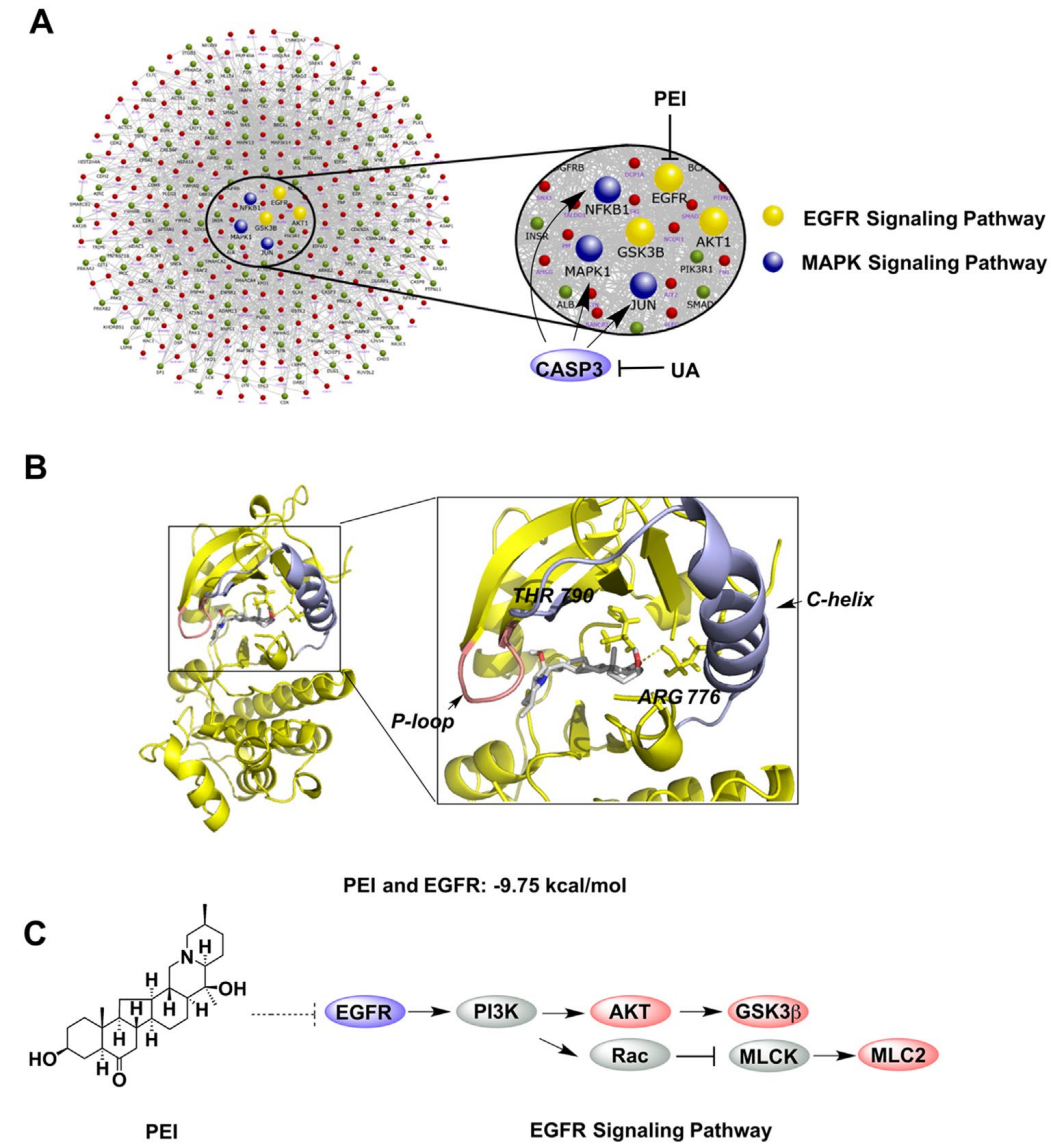
Bioinformatics analyses for screening the key bioactive compound and predicting its target and signaling pathway. **(A)** The PPI analysis of the phosphorylated proteins related to the key node and function. **(B)** Molecular docking of PEI with EGFR; PyMOL software was used to display the 3D map of the interaction of PEI with EGFR (PDB:3W2S). **(C)** The bioinformatics pathway analysis was used to predict that the bioactive ingredient PEI acted on the EGFR signaling pathway.

To further predict the key pathways on which CBPP acted, PPI analysis was used on the phosphorylated proteins related to the cytoskeleton and inflammation. As **Figure 3A** shows, there were a total of 341 proteins that interacted with the phosphorylated proteins, and the phosphorylated proteins were regulated by the six focused proteins in the protein–protein interaction network. The six focused proteins could be divided into MAPK and EGFR signaling pathways. The results suggest that CBPP mainly affects these two pathways to alleviate COPD. In our previous study, ursolic acid (UA) and PEI were identified as the main compounds of CBPP, and we have illustrated that UA affects MAPK signaling pathways by acting on CASP3 (Ma et al., 2017). Moreover, PEI has the potential to inhibit EGFR in EGFR signaling pathway. To analyze the interactions of PEI with EGFR (PDB:3W2S), molecular docking was used for further prediction. We then analyzed the top-scoring poses of PEI in all cases, which are displayed as 3D maps (**Figure 3B**). The hydroxyl of PEI interacted with Thr-790 and Arg-776 of EGFR. Meanwhile, the binding energy of PEI with EGFR was low. This finding suggested that PEI may act on EGFR. Thus, we integrated the iTRAQ quantitative, bioinformatics analysis, molecular docking, and the previous results to predict that PEI is the main ingredient that acts upon EGFR signaling pathways to improve the condition of lung cells. The signaling pathway on which PEI acts was predicted, as shown in **Figure 3C**. This prediction suggested that the potential activities of PEI were improving lung function and anti-fibrosis effects.

### PEI Activates MLC2 and Improves Lung Function

**Figure 4:**
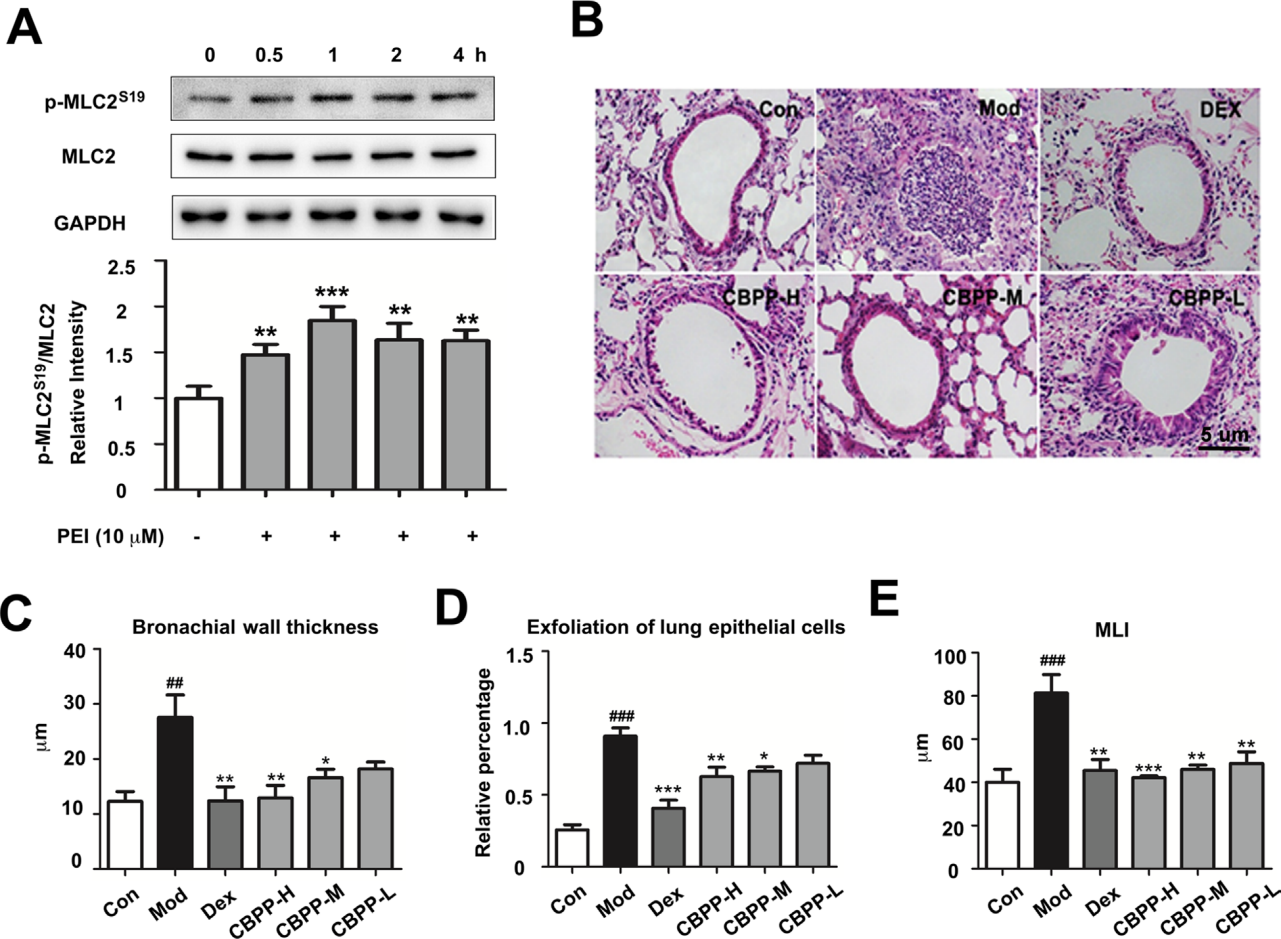
PEI and CBPP increased phosphorylation of MLC2 and improved lung function. **(A)** HBSMC cells were stimulated by 10 µM PEI, phosphorylation of MLC2 was measured by Western blot, and the relative intensity data of P-MLC2^S19^ to GAPDH are represented by the mean ± SD of three groups, **p < 0.01 and ***p < 0.001. **(B)** HE staining images (400-fold) of bronchus, the statistics of bronchial wall thickness **(C)**, exfoliation of lung epithelial cells **(D)**, and MLI **(E)** in bronchus pathological section. *p < 0.05, **p < 0.01, and ***p < 0.001 compared to the Mod. ^##^ *p* < 0.01 and ^###^ *p* < 0.001, compared to the Con (n = 6).

To verify the PPI analysis results, the effect of PEI on MLC2 was detected by Western blot. As shown in **Figure 4A**, Western blot analysis revealed that the phosphorylation of MLC2^S19^ was markedly increased by PEI treatment in HBSMC cells at different time points compared to the control group. This result is consistent with the phosphoproteomics and bioinformatic analyses. Based on this finding, we next investigated the effect of CBPP on the lung function *in vivo*. As shown in **Figure 4B**, compared to the Con group, smog stimulation caused bronchial wall thickness (**Figure 4C**), exfoliation of lung epithelial cells (**Figure 4D**), and an increase in the pulmonary mean linear intercept (MLI) (**Figure 4E**). Meanwhile, treatment with CBPP significantly reduced the histologically detectable injury in a dose-response manner. This result suggested that smog stimulation caused a decrease in lung function and that CBPP could improve lung function in COPD.

### PEI Inhibits the EGFR Signaling Pathway and Activates Pulmonary Fibrosis

**Figure 5:**
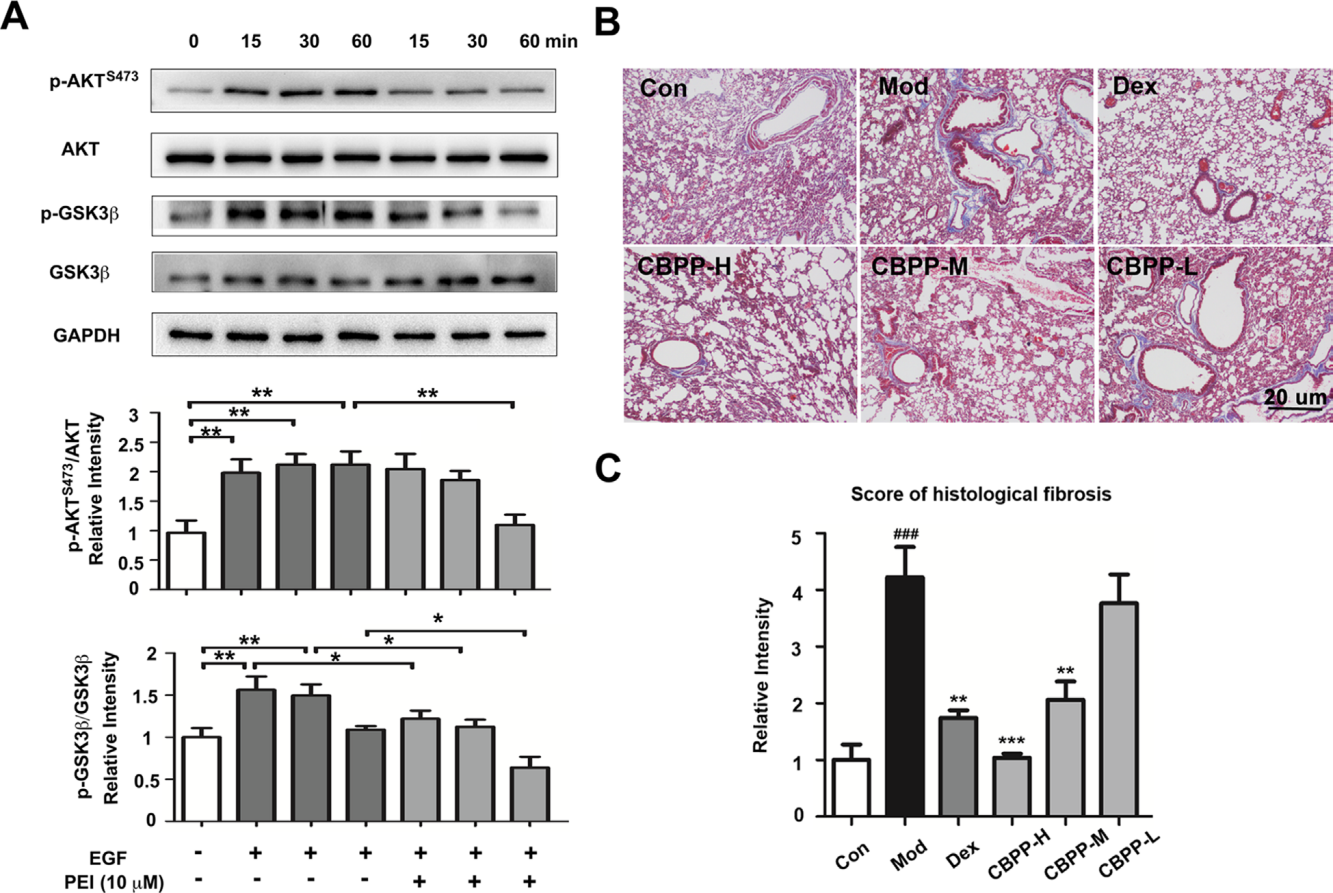
PEI inhibited the EGFR signaling pathway, and CBPP alleviated pulmonary fibrosis. **(A)** BEAS-2B cells were pretreatment with PEI, then stimulated by 1 ng/ml EGF. The phosphorylation of AKT^S473^ and GSK3β was measured by Western blot, and the relative intensity data of P-AKT^S473^ and P-GSK3β to GAPDH are represented by the mean ± SD of three groups, *p < 0.05 and **p < 0.01. **(B)** Masson staining images (40-fold) of lung pathological sections and **(C)** statistics for the score of histological fibrosis, **p < 0.01 and ***p < 0.001 compared to the Mod. ^###^ *p* < 0.001 compared to the Con (n = 6).

To verify the anti-fibrosis effects of PEI and CBPP, the effects of PEI on the EGFR signaling pathway in HBSMC cells were measured by Western blot. The phosphorylation of AKT^S473^ is regulated by EGFR, and it is a biomarker in pulmonary fibrosis (Korfhagen et al., 2009). As shown in **Figure 5A**, the phosphorylation of AKT^S473^ and GSK3β was increased in the EGF-treated group at 15, 30, and 60 min. However, the phosphorylation of AKT^S473^ was markedly inhibited at 15, 30, and 60 min, and the phosphorylation of GSK3β was markedly inhibited at 30 and 60 min, by PEI. The result is consistent with the prediction and suggests that PEI has anti-fibrosis effects by inhibiting EGFR. To verify the result *in vivo*, we next detected the histological changes in pulmonary fibrosis; as shown in **Figure 5B and C**, the deposition of collagen fibers was significantly increased in the smog stimulation group and was decreased after treatment with CBPP in a dose-response manner. This finding suggests that CBPP relieves the pulmonary fibrosis caused by chemical stimulation in COPD.

## Discussion

It is increasingly recognized that bioactive compounds might influence chronic respiratory diseases. Sulforaphane has the active effect to the transcription factor Nrf2, which is reduced in the lungs of COPD patients, and the deficiency causes inflammation and corticosteroid resistance (Wise et al., 2016). Resveratrol has the anti-inflammatory effect in the treatment of COPD that acts by attenuating the release of inflammatory cytokines but is poorly bioavailable (Knobloch et al., 2014). Moreover, celastrol may attenuate COPD by inhibiting inflammation development by suppressing the Ednrb/Kng1 signaling pathway (Shi et al., 2018). Hence, it is very necessary to find active natural products to prevent early stage COPD from natural products. In this paper, phosphoproteomics technology was used to screen the classic prescription of CBPP, with the hope of providing a solution for identifying the key bioactive compounds and revealing its mechanisms.

In COPD, chronic inflammation and structural remodeling are pivotal pathological features caused, in part, by the aberrant function of airway smooth muscle (Perry et al., 2018). Myosin light chain 2 (MLC2) is a diagnostic marker in COPD, and its phosphorylation plays key roles in the activation of actin and myosin motor to provide the contractility for several cellular processes in smooth muscle cells, such as cell contraction, stress fiber formation, cell motility, and cytokinesis (Duan et al., 2016). The improvement of the phosphorylation level of MLC2 is necessary for enhancing lung function. PEI is the main component of *Fritillaria*, and it has an effect to be an antitussive and a relaxant of bronchial smooth muscle (Wang et al., 2012). In our study, we found that PEI increased the phosphorylation of MLC2^S19^ by inhibiting EGFR in HBSMC, which could promote the contraction of smooth muscle and lung function.

In previous reports, fibrosis was observed in the lungs of COPD patients, predominately around small airways, and small airway fibrosis and obliteration are associated with the lung function decreasing. EGF is a ligand for EGFR, and activation of EGFR is related to pulmonary fibrosis in lung disease (Korfhagen et al., 2009). Moreover, the activation of the PI3K/Akt signaling pathway was regulated by EGFR-induced pulmonary fibrosis (Zhang et al., 2016). Thus, acting on the EGFR/PI3K/AKT signaling pathway is essential for the treatment of pulmonary fibrosis in COPD. PEI has an effect of alleviating the lung injury, which is bleomycin-induced in rats and of alleviating pulmonary fibrosis, which was associated with a reduction in the levels of **connective tissue growth factor** (CTGF), TGF-β, NF-κB, ERK1/2, and tumor necrosis factor ligand superfamily member 6 (FasL) in lung tissue (Guo et al., 2013). Nevertheless, few studies have stated the molecular mechanism of PEI in pulmonary fibrosis. In our study, EGF triggers the EGFR signaling pathway, which may induce fibrosis, and PEI can inhibit the activation of downstream targets of EGFR, such as AKT and GSK3β. This finding suggested the potential mechanism by which PEI exerts its anti-fibrosis effect by acting on EGFR on the EGFR signaling pathway. In addition, few bioactive compounds have been reported that have anti-fibrosis effects in the treatment of COPD at present. The results in this study may reveal a new bioactive compound for early intervention in pulmonary fibrosis.

Studies have shown that the levels of activation of the p38 MAPK, JNK1/2, and ERK1/2 on MAPK signaling pathways are increased in patients with COPD (Lemire et al., 2012). This activation is because the MAPK signaling pathway is activated by the stimulation of cigarette smoke and air pollution and has increased activity in COPD alveolar macrophages. In addition, the MAPK signaling pathway is involved in the regulation of the synthesis of inflammation mediators at the level of transcription and translation, making it a potential target for anti-inflammatory therapeutics. Moreover, although the center of pulmonary fibrosis is cell death, the macrophage polarization that is led by the proper cytokine environment is critical too (Homer et al., 2011). Stefan et al. reported that inflammation leads to tissue injury and fibrosis by the expression of IL-13 (Fichtner-Feigl et al., 2006). Hence, IL-13 is critical for relieving inflammation in stage I of COPD. UA is the main anti-inflammatory compound in CBPP and is widely distributed in many plants. UA has been proven to have the potential for alleviating inflammation-associated diseases in clinical tests (Ikeda et al., 2008). Studies reported that UA has the effect to inhibit phosphorylation of MEK and c-Raf, and the downstream targets, such as ERK and JNK, which were induced by mitogen in lymphocytes (Kaewthawee and Brimson, 2013). In our previous study, our results suggested that UA reduced the cytokines and alleviated inflammation disorders *via* inhibiting CASP3. In this study, the analyses of phosphoproteomics and PPI are consistent with previous research and suggest that UA has the effect of alleviating inflammation by inhibiting CASP3. Moreover, PEI has the effect of decreasing the expression of NF-κB in our previous study, and it may be due to the inhibition on the phosphorylation of AKT, which was the upstream target of NF-κB. It suggested that PEI and UA may be synergistic in the treatment of inflammation, but it needs to be further verified.

COPD is a complex disease and is only partially reversible; thus, the prevention of the exacerbation of COPD in the early phase is crucial. In this study, we identified the key bioactive compounds from CBPP by phosphoproteomics analysis and provided a solution for analysis and identification of the main natural products and bioactive compounds from a compound mixture. At the same time, the results suggested the potential of UA and PEI as therapeutic agents for preventing the exacerbation of COPD by acting on the MAPK, EGFR, and MLC2 signaling pathways.

## Data Availability Statement

The datasets generated for this study can be found in www.iprox.org, IPX0001570000.

## Ethics Statements

This study was carried out in accordance with the recommendations of the Principle of Laboratory Animal Care (NIH Publication no. 85-23, revised 1985) guidelines, Animal Ethics Committee of Nankai University. The protocol was approved by the Animal Ethics Committee of Nankai University (TCM-LAEC2016031).

## Author Contributions

GB and YH designed the study. XM performed experiments, acquired and analyzed the data, and drafted and edited the manuscript. AL, WL, ZW, and NC assisted with the experiments. SL and JL contributed to the discussion and review of the manuscript.

## Funding

This study was supported by the National Key Research and Development Program of China (No. 2018YFC1704800, 2018YFC1704805), National Natural Science Foundation of China (No. 81673616), International Cooperation and Exchange of the National Natural Science Foundation of China (No. 81761168039), and the Fundamental Research Funds for the Central Universities, Nankai University (No. 63191723).

## Conflict of Interest Statement

The authors declare that the research was conducted in the absence of any commercial or financial relationships that could be construed as a potential conflict of interest.

The reviewer MJ declared a shared affiliation, with no collaboration, with several of the authors, XM, WL, ZW, NC, YU, GB, to the handling editor at time of review.

## References

[B1] Alayev A. Doubleday P. F. Berger S. M. Ballif B. A. Holz M. K. (2014). Phosphoproteomics reveals resveratrol-dependent inhibition of Akt/mTORC1/S6K1 signaling. J. Proteome Res. 13, 5734–5742. 10.1021/pr500714a 25311616 PMC4258159

[B2] Beran D. Zar H. J. Perrin C. Menezes A. M. Burney P. (2015). Burden of asthma and chronic obstructive pulmonary disease and access to essential medicines in low-income and middle-income countries. Lancet Respir. Med. 3, 159–170. 10.1016/S2213-2600(15)00004-1 25680912

[B3] Changxiao L. (2018). Understanding “medicine and food homology”, developing utilization in medicine functions. Chin Herb. Med. 10, 337–338. 10.1016/j.chmed.2018.10.006

[B4] Duan X. Liu J. Zhu C. C. Wang Q. C. Cui X. S. Kim N. H. (2016). RhoA-mediated MLC2 regulates actin dynamics for cytokinesis in meiosis. CELL CYCLE. 15, 471–477. 10.1080/15384101.2015.1128590 26701676 PMC4943703

[B5] Fichtner-Feigl S. Strober W. Kawakami K. Puri R. K. Kitani A. (2006). IL-13 signaling through the IL-13a2 receptor is involved in induction of TGF-b1 production and fibrosis. Nat. Med. 12, 99–106. 10.1038/nm1332 16327802

[B6] Girolamo G. Petsalaki E. (2017). Proteomics and phosphoproteomics in precision medicine: applications and challenges. Brief. Bioinformatics ra15–ra15, 1–11. 10.1093/bib/bbx141 PMC6585152 29077858

[B7] Guo H. Ji F. Liu B. Chen X. He J. Zhao X. (2013). Peiminine ameliorates bleomycin-induced acute lung injury in rats. Mol. Med. Rep. 7, 1103–1110. 10.3892/mmr.2013.1312 23404624

[B8] Hoffmann B. Luttmann-Gibson H. Cohen A. Zanobetti A. de Souza C. Foley C. (2012). Opposing effects of particle pollution, ozone, and ambient temperature on arterial blood pressure. Environ. Health Perspect. 120, 241–246. 10.1289/ehp.1103647 22020729 PMC3279434

[B9] Homer R. J. Elias J. A. Lee C. G. Herzog E. (2011). Modern concepts on the role of inflammation in pulmonary fibrosis. Arch. Pathol. Lab. Med. 135, 780–788. 10.1043/2010-0296-RA.1 21631273

[B10] Ikeda Y. Murakami A. Ohigashi H. (2008). Ursolic acid: an anti- and pro-inflammatory triterpenoid. Mol. Nutr. Food Res. 52, 26–42. 10.1002/mnfr.200700389 18203131

[B11] Jin Tao Y. H. Ma X. Liu D. Tong Y. Zhou H. Zhou H. (2016). An integrated global chemomics and system biology approach to analyze the mechanisms of the traditional chinese medicinal preparation eriobotrya japonica -fritillaria usuriensis dropping pills for pulmonary diseases. BMC Complement. Altern. Med. 16, 4. 10.1186/s12906-015-0983-y 26742634 PMC4705596

[B12] Kaewthawee N. Brimson S. (2013). The effects of ursolic acid on cytokine produce via the MAPK pathways in leukemic T-cells. EXCLI J 12, 102–114. 10.17877/DE290R-10658 26417220 PMC4531796

[B13] Knobloch J. Wahl C. Feldmann M. Jungck D. Strauch J. Stoelben E. (2014). Resveratrol attenuates the release of inflammatory cytokines from human bronchial smooth muscle cells exposed to lipoteichoic acid in chronic obstructive pulmonary disease. Basic Clin. Pharmacol. Toxicol. 114, 202–209. 10.1111/bcpt.12129 23981542

[B14] Ko F. W. Hui D. S. (2012). Air pollution and chronic obstructive pulmonary disease. Respirology 17, 395–401. 10.1111/j.1440-1843.2011.02112.x 22142380

[B15] Korfhagen T. R. Le Cras T. D. Davidson C. R. Schmidt S. M. Ikegami M. Whitsett J. A. (2009). Rapamycin prevents transforming growth factor-a–induced ulmonary fibrosis. Respir. Cell Mol. Biol. 41, 562–572. 10.1165/rcmb.2008-0377OC PMC2778163 19244201

[B16] Lemire B. B. Debigaré R. Dubé A. Thériault M. E. Côté C. H. (2012). MAPK signaling in the quadriceps of patients with chronic obstructive pulmonary disease. J Appl Physiol. 113, 159–166. 10.1152/japplphysiol.01518.2011 22518834

[B17] Locard-Paulet M. Lim L. Veluscek G. McMahon K. Sinclar J. van Weverwijk A. (2016). Phosphoproteomic analysis of interacting tumor and endothelial cells identifies regulatory mechanisms of transendothelial migration. Science Signal 9 (414), ra15. 10.1126/scisignal.aac5820 PMC6485367 26861043

[B18] López-Campos J. L. Tan W. Soriano J. B. (2016). Global burden of COPD. Respirology 21, 14–23. 10.1111/resp.12660 26494423

[B19] Ma J. Zheng J. Zhong N. Bai C. Wang H. Du J. (2018). Effects of YuPingFeng granules on acute exacerbations of COPD: a randomized, placebo-controlled study. Int. J. COPD. 13, 3107–3114. 10.2147/COPD.S170555 PMC6174891 30323581

[B20] Ma X. Zhang Y. Wang Z. Shen Y. Zhang M. Nie Q. (2017). Ursolic acid, a natural nutraceutical agent, targets caspase3 and alleviaes inflammation-associated downstream signal transduction. Mol. Nutr. Food Res. 61, 1700332. 10.1002/mnfr.201700332 PMC5765441 28801966

[B21] Mohamed Ismail A. A. Ling Eng T. Wu D. B. C. Fiona P. Gerald Seng W. C. Liang L. (2018). Comparative efficacy of inhaled medications (ICS/LABA, LAMA, LAMA/LABA and SAMA) for COPD: a systematic review and network meta-analysis. Int. J. COPD. 13, 3203–3231. 10.2147/COPD.S173472 PMC6186767 30349228

[B22] Norebert B. (2016). Contribution of air pollution to COPD and small airway dysfunction. Respirology. 21, 237–244. 10.1111/resp.12644 26412571

[B23] Parker R. Vella L. J. Xavier D. Amirkhani1 A. Parker J. Cebon J. (2015). Phosphoproteomic analysis of cell-based resistance to BRAF inhibitor therapy in melanoma. Front Oncol. 5, 95. 10.3389/fonc.2015.00095 26029660 PMC4432663

[B24] Perry M. M. Tildy B. Papi A. Casolari P. Caramori G. Remple K. L. (2018). The anti-proliferative and anti-inflammatoryresponse of COPD airway smooth musclecells to hydrogen sulfide. Respir. Res. 19, 85. 10.1186/s12931-018-0788-x 29743070 PMC5944010

[B25] Shi K. Chen X. Xie B. Yang S. Liu D. Dai G. (2018). Celastrol alleviates chronic obstructive pulmonary disease by inhibiting cellular inflammation induced by cigarette smoke via the Ednrb/Kng1 signaling pathway. Front Pharmacol. 9, 1276. 10.3389/fphar.2018.01276 30498444 PMC6249343

[B26] Singh D. Maleki-Yazdi M. R. Lee T. Ahmar I. Fahy W. A. Naya I. (2016). Prevention of clinically important deteriorations in COPD with umeclidinium/vilanterol. Int. J. COPD. 11, 1413–1424. 10.2147/COPD.S101612 PMC4928660 27445468

[B27] Tashkin D. P. Fabbri L. M. (2010). Long-acting β-agonists in the management of chronic obstructive pulmonary disease: current and future agents. Respir. Res. 11, 149–63. 10.1186/1465-9921-11-149 PMC2991288 21034447

[B28] Wang D. Wang S. Chen X. Xu X. Zhu J. Nie L. (2012). Antitussive, expectorant and anti-inflammatory activities of four alkaloids isolated from Bulbus of Fritillaria wabuensis. J. Ethnopharmacol. 139, 189–193. 10.1016/j.jep.2011.10.036 22101082

[B29] Wang Z. Kim U. Jiao Y. Li C. Guo Y. Ma X. (2019). Quantitative proteomics combine with affinity MS revealedthe molecular mechanism of ginsenoside anti-tumor effects. J. Proteome Res. 2100–2108. 10.1021/acs.jproteome.8b00972 30860844

[B30] Wei W. Shin Y. S. Xue M. Matsutani T. Masui K. Yang H. (2016). Single-cell phosphoproteomics resolves adaptive signaling dynamics and informs targeted combination therapy in glioblastoma. Cancer Cell 29 (4), 563–573. 10.1016/j.ccell.2016.03.012 27070703 PMC4831071

[B31] Wellenius G. A. Burger M. R. Coull B. A. Schwartz J. Suh H. H. Koutrakis (2012). Ambient air pollution and the risk of acute ischemic stroke. Arch. Intern. Med. 172, 229–234. 10.1001/archinternmed.2011.732 22332153 PMC3639313

[B32] Wise R. A. Holbrook J. T. Criner G. Sethi S. Rayapudi S. Sudini K. (2016). Lack of effect of oral sulforaphane administration on Nrf2 expression in COPD: a randomized, double-blind, placebo controlled trial. PLoS ONE 11 (11), e0163716. 10.1371/journal.pone.0163716 27832073 PMC5104323

[B33] Zhang X. L. Xing R. G. Chen L. Liu C. R. Miao Z. G. (2016). PI3K/Akt signaling is involved in the pathogenesis of bleomycin-induced pulmonary fibrosis via regulation of epithelial-mesenchymal transition. Mol. Med. Rep. 14 (6), 5699. 10.3892/mmr.2016.5960 27878273

